# Understanding public service supply chain management: a systematic literature review

**DOI:** 10.1007/s11301-023-00350-8

**Published:** 2023-05-22

**Authors:** Katarzyna Sienkiewicz-Małyjurek, Maciej Szymczak

**Affiliations:** 1 Department of Management, Faculty of Organization and Management, Silesian University of Technology, Roosevelta 26 Str., 41-800 Zabrze, Poland; 2 Vice-Rector for Development and External Relations, Poznań University of Economics and Business, Niepodległości 10 Avenue, 61-875 Poznań, Poland

**Keywords:** Supply chain management, Service supply chain, Public service logic, Relationship management, Public service supply chain management (PSSCM), Systematic literature review, H4, H7, R5

## Abstract

The complexity of delivering public services under dynamically changing operating conditions causes uncertainty in those processes. Economic and social crises, pandemics, natural and technological threats and local armed conflicts add more layers of complexity and force governments to seek ways to ensure the continuous supply of these services. Therefore, researchers indicate that a supply chain management approach could increase the efficiency and quality of public service implementation processes. However, the scattered research that exists on this topic occurs in limited areas of public governance. Therefore, this paper aims to understand the importance of supply chain management in public service delivery processes, develop the public service supply chain model, analyse the evolution of the research and identify research streams in this exploratory area. A systematic literature review based on the PRISMA methodology serves to achieve this purpose. The paper defines public service supply chain management as a synchronised process of co-creating value in public networks with its basis in relationship management, in which each actor can be both a supplier and an end-user. This paper also presents a bibliographic visualisation of research issues in public service supply chain management (PSSCM) and identifies eight major research streams in this area.

## Introduction

Services dominate modern social and economic life. They are the main source of GDP growth and prosperity in every modern society, and as a result, their practical and scientific importance continues to increase (Li and Qiu 2020). During the last few years, scientists have endeavoured to understand and treat public service delivery processes from a service-dominant logic perspective. These have included attempts to shift from a market orientation towards user-oriented public services (Powell and Osborne 2020; Hodgkinson et al. 2017) to perceiving public service delivery processes as networks (Klijn and Koppenjan 2016) or ecosystems (Petrescu 2019; Jaakkola et al. 2015). However, public-sector research on issues of service management is still in its infancy, although public expenditure on services is several times greater than that of private organisations and individuals (Osborne et al 2021; Hodgkinson et al. 2017). In addition to the emergence of public service management, supply chain research in the context of the public sector has begun to develop (Seepma et al. 2020; Arlbjørn et al. 2011; Callender 2011). The potential of using the principles of supply chain management in the public sector to address the need to co-create public value is increasingly apparent (Seepma et al. 2020), and the need for greater research into public service supply chain management (PSSCM) continues to grow (Engen et al. 2020; Powell and Osborne 2020; Petrescu 2019; Hodgkinson et al. 2017).

This paper develops PSSCM achievements by addressing two research gaps. First, reviewing the activities that service supply chains perform aims to categorise individual organisations’ resources as primary and ancillary services that create user value (Baltacioglu et al. 2007; Hochrein et al. 2015). The foundations of service supply chains and the principles of their operation support the co-creation of values (Akyuz and Gursoy 2020). However, compared to the private sector, less research on supply chain management in the public sector exists (Harland et al. 2019; Callender 2011), and it primarily adopts the perspective of organisational effectiveness rather than investigating the quality of the public services the organisations deliver. Some scholars believe that ‘it remains a misconstrued and unappreciated domain’ (Mafini 2016, p. 256). Assessing the performance of public service delivery (Karaba et al. 2022; Chorfi et al. 2018; Fan et al. 2017) requires supply chain management research that ‘can both explore and change policy’ (Harland et al. 2019, p. 6). In addition, the research should emphasise the need for a holistic network approach to supply chains in the public sector over implementing individual services in dyads (Radnor Noke 2013; Gianakis and McCue 2012). Studies that refer to public supply chains holistically are rare. Instead, researchers focus on specific fields (e.g. health care or humanitarian aid) or forms (public–private partnership and public procurement) without considering contemporary paradigms of public governance. This paper contributes to closing this research gap by comprehensively defining PSSCM and proposing a model for it. Second, supply chains in the public sector have a specific complex nature, and their processes are subject to normative, political and social pressures (Esain et al. 2016). Callender (2011, p. 9) notes that the ‘absence of alignment of supply chains linking the activities of public agencies’ works to the detriment of chain stakeholders. However, the extent to which implementation of best practices associated with supply chain management characterises public service delivery processes remains unclear (Harland et al. 2019; Mafini 2016). Therefore, a research gap exists regarding the possibility of using supply chain management to better understand and conduct public service delivery processes from the perspective of public service logic. Therefore, this paper identifies research streams in this exploratory area, seeking to better understand the specific uses of supply chain management in public service delivery processes by answering the following research questions:

1. What is PSSCM?
2. How is PSSCM structured?
3. How has the evolution of research on PSSCM progressed?
4. What research is conducted on PSSCM?

This study uses scientific outputs in public service logic theory and service supply chain management to investigate the specificity of PSSCM. A systematic literature review (SLR) of service supply chain management in the most representative public services supports identifying the nature of PSSCM. The investigations in this paper also reveal the main research streams within PSSCM. The results advance the research by defining PSSCM, elaborating on its model and systematising knowledge of functioning supply chains in the public sector. These findings could aid decision-makers in meeting the needs of public service stakeholders.

The remainder of the paper is organised as follows. First, the theoretical foundation section reviews the relevant literature on the public service delivery process, public service logic theory and the nature of PSSCM. The methodology section elaborates on the research, and a presentation of the findings follows. Finally, a discussion of theoretical contributions and managerial implications, limitations and future research directions conclude the paper.

## Theoretical foundations

### Public services delivery

Services are generally ‘the application of specialized competences (knowledge and skills), through deeds, processes, and performances for the benefit of another entity or the entity itself’ (Lusch and Vargo 2006, p. 283). In this approach, public services are ‘services provided to the public to address social or economic ills or issues, whether provided by the public, private, or third (non-profit) sectors’ (Osborne 2021, p. 20). Differentiating types of public services can produce the following list: general, defence, public order and safety, economic affairs, environmental protection, housing and community amenities, health, recreation, culture and religion, education and social protection (COFOG 2019). The delivery of such services is complex and involves actions within the framework of interorganisational and intersectoral collaboration to provide these services as society expects.

The complexity of public services delivery derives from its diversity, the diversification of social needs, structural changes, social inequality, economic pressure and legal and organisational regulations. Therefore, public service delivery processes require multidimensional solutions (COFOG 2019; Methodological Framework of the Principles of Public Administration 2019). Due to this complexity, public administration, which is responsible for the provision of public services, cannot individually implement the statutory tasks the responsibility imposes on it. As a result, the mechanisms that incorporate private and nongovernmental organisations in delivering public services are continuously developing. These include public procurement, public–private partnerships, outsourcing and social contracts (Aben et al. 2021; Caldwell et al. 2017; Amann et al. 2014; Zheng et al. 2008). Furthermore, governments no longer play dominant roles in public service delivery processes but are network orchestrators that provide the conditions for societal development and self-organisation towards the co-creation of public value (Li and Qiu 2020; Osborne 2021). Each of a network’s entities may have different, sometimes contradictory or divergent needs for a given public service, increasing the complexity of public service delivery processes. As a result, the embedding of contemporary public service delivery processes in complex intersectoral networks leads to relationships developing into complex ecosystems, in line with service management theory (Hodgkinson et al. 2017; Jaakkola et al. 2015).

According to Grönroos (1990, p. 7), service management is about understanding ‘the utility or value customers receive by consuming or using the offerings of the organisation and how services alone or together with physical goods or other kinds of tangibles contribute to this utility’. Understanding how perceived value changes over time and whether organisations are prepared to cope with these changes is also important. Generally, ‘value creation is at the core of management research and practice’ (Caldwell et al. 2017, p. 906). In the public sector, service management focuses on entities’ efforts to provide these services and co-create public value (Osborne 2021; Aben et al. 2021; Hodgkinson et al. 2017). Each of these entities plays a specific role, necessary for the co-creation of public value; they often also decide whether co-creation occurs. This approach to the provision of public services is under development.

Hartley et al. (2017, p. 671) states that the interpretation of public value is as a ‘contribution to the public sphere, […] the addition of value through actions in an organizational or partnership setting and the heuristic framework of the strategic triangle (the public value proposition, the authorizing environment and operational resources which a public manager has to align to achieve public value)’. Collaborative networks and ecosystems are the sites for creating public value, through the interactions of the many entities involved in public service delivery processes (Karaba et al. 2022; Osborne 2021). These entities also include service users. Therefore, all entities in a given public service delivery process co-create public value (Caldwell et al. 2017; Kivleniece and Quelin 2012). In this approach, the public decides what public services governments should offer and how they should create these services, the basis of public service logic theory.

### The basis of public service logic theory

Until recently, the public sector was the main provider of public services, and research in this area assumed an orientation towards internal costs and good-dominant logic (Osborne 2018; Hodgkinson et al. 2017). This approach may lead to increased transparency in decision-making and efficiency in public service delivery. However, its practical disadvantages limit accountability, sustainable development and equality while increasing the gaps between public services, social needs and the opportunism of public service providers (Li and Qiu 2020), by failing to consider external efficiency and co-creation of public value (Powell and Osborne 2020).

Currently, public service providers postulate user orientation and relationship management in intersectoral networks with the inseparability and heterogeneity of public services (Li and Qiu 2020; Vargo and Lusch 2008). The development of value creation theory in the public sector is consistent with service-dominant logic, where the value creation process arises through mutual interactions and resource integration. According to this approach, skills and knowledge are key resources exchanged within collaborative networks (Vargo et al. 2008; Payne et al. 2008). Value creation results from the coordinated actions of entities that are both the suppliers and the users of a service. This process does not focus on implementing individual organisations’ basic processes but results from the dynamic exchange of values between them, the foundation on which public service logic theory develops (Engen et al. 2020; Osborne 2018).

Public service logic theory refers to the interaction between actors involved in the public service delivery process and sharing their resources to co-create value (Osborne 2018; Grönroos 2011). According to Osborne (2018), this approach departs from the focus on the efficiency and effectiveness of the public service delivery process and takes value as a key indicator for public service evaluation. It indicates that a service provider can only offer value as interactions between service users, public sector organisations and other entities in the public service delivery process create it. The main difference between this and the previous paradigms of public governance (e.g. New Public Management) lies in the roles of public service users and public sector organisations. In public service logic theory, the user co-creates public services through interactions with ‘the provider in mutual exchanges, during use, consumption or experience’ (Petrescu 2019, p. 1734). Users also contribute to co-creating public value through their knowledge, ideas and time.

Public service logic theory emphasises that the creation and consumption of a public service occur in parallel (Hodgkinson et al. 2017). The creation of private value (that an individual uses) and of group and public value (that a group of inhabitants and society overall use, respectively) (Engen et al. 2020; Petrescu 2019) occurs all at once. For example, by responding to a robbery, the police help the victim, and the victim’s acceptance of the police’s help creates value for the victim. At the same time, the police indicate that they control the level of security in a given district with their actions. Group value is co-created when the police interact with the inhabitants of the district to establish the facts and circumstances of a robbery. By gaining new experience, the police increase their competency, which they use to create public value for society as a whole. Within this framework, each actor can simultaneously play different roles in the process of co-creating value (Engen et al. 2020; Petrescu 2019). For example, every citizen can be a user, volunteer, entrepreneur and employee of a public service organisation, all at once. Private sector organisations can simultaneously be providers and users of public services. Thus, the co-creation of public value has layers, and the need to integrate all actors (often with conflicting interests), as well as various roles and resources, arises. As a result, user orientation and the integration of actors and their resources on many levels pave the way to implement PSSCM, to develop the current public governance paradigm.

### The nature of supply chain management in public service delivery

The traditional approach to supply chain management concerns material flows, but the development of service orientation in this area has been apparent for more than 20 years (Akyuz and Gursoy 2020; Arlbjørn et al. 2011; Baltacioglu et al. 2007; Ellram et al. 2004). According to Baltacioglu et al. (2007, p. 112), a service supply chain could be ‘the network of suppliers, service providers, consumers and other supporting units that performs the functions of transaction of resources required to produce services; transformation of these resources into supporting and core services; and the delivery of these services to customers’. In such chains, flows are intangible, heterogeneous and volatile, distinguishing them from product supply chains (Kalra et al. 2021; Arlbjørn et al. 2011; Baltacioglu 2007; Ellram et al. 2004). However, flow intangibility applies strictly to service provision and the use of adequate skills and knowledge. According to Aitken et al. (2016), many service processes have tangible results, e.g. repairing a car after a collision has a tangible effect. In the public sector, the delivery of services also has a tangible effect. For example, delivering water to consumers occurs when customers need and use water at the place, quantity, price and quality they choose. The physical flow of water is auxiliary to the process of providing the service. Moreover, from the service perspective, the supply chain input and output, the ‘push and pull’ strategy, customer relations and offer portfolios are also important (Mazzawi and Alawamleh 2019; Aitken et al. 2016; Cohen et al. 2006).

In service supply chains, operations aim to transform the resources of individual organisations into basic services (e.g. media provision or medical advice) and auxiliary services (e.g. the maintenance of technical infrastructure or medical examinations) that create value for the customer. Service providers in such chains deal with constant needs that may fluctuate periodically and require the ability to meet surplus demand for services. However, as a result, they must also prepare for sudden, unpredictable events and the emergence of additional demands. Therefore, the management of service supply chains involves designing the service provision process and combining the roles, resources, knowledge and skills of various actors to jointly create high-quality service (Howard et al. 2017; Callender 2011; Ellram et al. 2004). It entails ‘the management of information, processes, capacity, service performance and funds from the earliest supplier to the ultimate customer’ (Ellram et al. 2004, p. 25).

Service supply chain management differs significantly between the private and public sectors (Seepma et al. 2020; Arlbjørn et al. 2011). From a public sector perspective, the goal of service supply chain management is to deliver high-quality public services, not to profit from selling services. Public service supply chains operate within the scope that legal regulations establish and implement their initiatives through taxes and fees, as well as public involvement (e.g. voluntary work). Private service supply chains operate through business models that they implement with hired labour and the profits they generate. Public service supply chain functions also entail transparency in their processes. As a result, their structure and the relations between their links are open and implemented on the basis of applicable legal regulations, system solutions and partnership agreements. An important feature that distinguishes public from private service supply chains is also the publicness of the investments they make and the results they achieve. The differences between public- and private-sector supply chain management and the nature of supply chain management in public service delivery make it a very promising and interesting research area.

Due to the described specificity of the functioning of supply chains in public service delivery processes, developing this research area seems beneficial and necessary. However, the first need is to understand and systematise this research area, which an SLR can successfully achieve.

## Methodology

This study used an SLR to achieve its research aims because reviews are ‘essential to advance the knowledge and understand the breadth of the research on a topic of interest, synthesize the empirical evidence, develop theories or provide a conceptual background for subsequent research, and identify the topics or research domains that require more investigation’ (Paré et al. 2015, p. 183). The SLR approach is a repeatable, transparent and rigorous publication review process that minimises research bias and errors (Tranfield et al. 2003; Kuckertz and Block 2021). Its methodology includes a standardised search and analysis framework that enables the collection and synthesis of relevant data from various knowledge bases, to gather comprehensive, objective material for analysis. The SLR identifies the current achievements in a specific field of knowledge on the basis of specific criteria (Clark et al. 2021; Fisch and Block 2018). It facilitates knowledge synthesis through gathering, analysing and verifying the current level of research. This SLR reflects the Preferred Reporting Items for Systematic Reviews and Meta-Analyses (PRISMA) Group methodology (Page et al. 2021).

This paper analyses the public services that appear most frequently in the supply chain literature. The initial literature review conducted at the beginning of the PRISMA framework indicates that the greatest amount of research addresses humanitarian aid (758 records found in Scopus and 783 records in Web of Science, by searching titles, abstracts and keywords), health care (2,218 records in Scopus and 776 in Web of Science), city management (2,842 in the Scopus and 1,880 in Web of Science), emergency management (2,388 records in Scopus and 1,823 in Web of Science) and general public services (446 records in Scopus and 308 in Web of Science). Concerning the classification of public services, according to COFOG (2019), research in the field of supply chain management also includes public safety (26 records in Scopus and 14 in Web of Science), defence (132 in Scopus and 113 in Web of Science), economic affairs (7 in Scopus and 3 in Web of Science), environmental management (528 records in Scopus and 94 in Web of Science), housing and community amenities (51 in Scopus and 39 in Web of Science), recreation (24 in Scopus and 14 in Web of Science), culture (3 in Scopus and 2 in Web of Science), education (369 in Scopus and 253 in Web of Science) and social care (18 in Scopus and 16 in Web of Science). These studies investigate the structure of supply chains, their impact on the effectiveness of delivering public services and the relations between actors in these chains. Given the number of publications in Scopus and Web of Science, this paper focuses on the most common public service research areas: humanitarian aid, health care, city management, emergency management and general public services. As these areas appear often in the literature, well-established and verified knowledge characterises them. In addition, public services, such as waste management, housing, community amenities, recreation and culture, qualify as ‘city management’, while many topics related to defence, public order and safety emerge from an emergency management perspective. Research also studies and applies issues of public safety and social protection in relation to humanitarian aid. Table 1 presents a comparison of city management, humanitarian aid, health care and emergency management, in terms of research and application.

**Table 1:** Comparison of city management, humanitarian aid, health care, and emergency management characteristics

Research area (differentiating factor)	City management	Humanitarian aid	Health care (public/non-public)	Emergency management
Level of consideration*	Meso	Meso	Meso and micro	Meso
Scope	City/city centre/urban areas	Local, regional, international	National/organisational, international	Local, regional, international
Nature of activity	Social	Social	Social/business	Social/business
Organisation, governance, funding	Public	Public	Public/private	Public
Goal	Quality of living and doing business	Rescue and help	Saving health and lives	Rescue and help
Economic drive	Cost minimisation, utility maximisation	Cost minimisation, utility maximisation	Cost minimisation, utility/profit maximisation	Utility maximisation, cost minimisation
System approach	Full ecosystem	Supply chain	Full ecosystem/supply chain	Supply chain
Network topology	Multi-node	Multi-echelon	Multi-echelon	Multi-echelon
Dominant research approach	Survey	Forecasting	Operations research	Forecasting
Research purpose	Synergy/collaboration	Optimisation	Optimisation	Optimisation/co-operation

*Micro: intra-organisational processes

Meso: inter-organisational relationship management

Macro: strategic aggregation of meso-level processes

Comparing the public governance fields (Table 1) that most often implement the concept of supply chain management reveals many similarities among them. Their application often occurs at the mesoeconomic level, and they relate to the social nature of supply chains, having public financing and an orientation towards cost minimisation and utility maximisation. Mesoeconomic strategies apply to relationships in the supply chain. In contrast, microlevel processes are internal to organisations, and macrolevel processes relate to regulations and the economic environment, aggregating processes occurring at the mesolevel. These similarities indicate that a holistic approach to PSSCM is possible.

The search for publications for the SLR occurred in August and September 2021, in Scopus and Web of Science, by combining the term ‘supply chain’ with the terms ‘public’, ‘healthcare’, ‘health care’, ‘humanitarian’, ‘emergency’ and ‘city’. The preliminary literature review was the basis for selecting these terms and identifying the most common research into the functioning of supply chains serving these governmental functions (COFOG 2019).

The selected keywords prioritise ‘supply chain’ and exclude ‘logistics’. Logistics includes single operations, while supply chain combines these operations, integrates interorganisational processes and incorporates elements that logistics does not consider, such as information systems, relationship management, trust and commitment (Cozzolino 2012; Lummus et al. 2001; Cooper et al. 1997). Moreover, supply chains are process-oriented and broader than logistics. In public service logic and public value co-creation, the process perspective is more applicable than the individual perspective (Cluley and Radnor 2021; Strokosch and Osborne 2020). According to Osborne (2021), public value arises in collaborative networks and ecosystems through cross-sectoral interactions. Therefore, to reflect the process and network orientation of public service delivery processes, this paper focuses on supply chains rather than logistics.

**Fig. 1:**
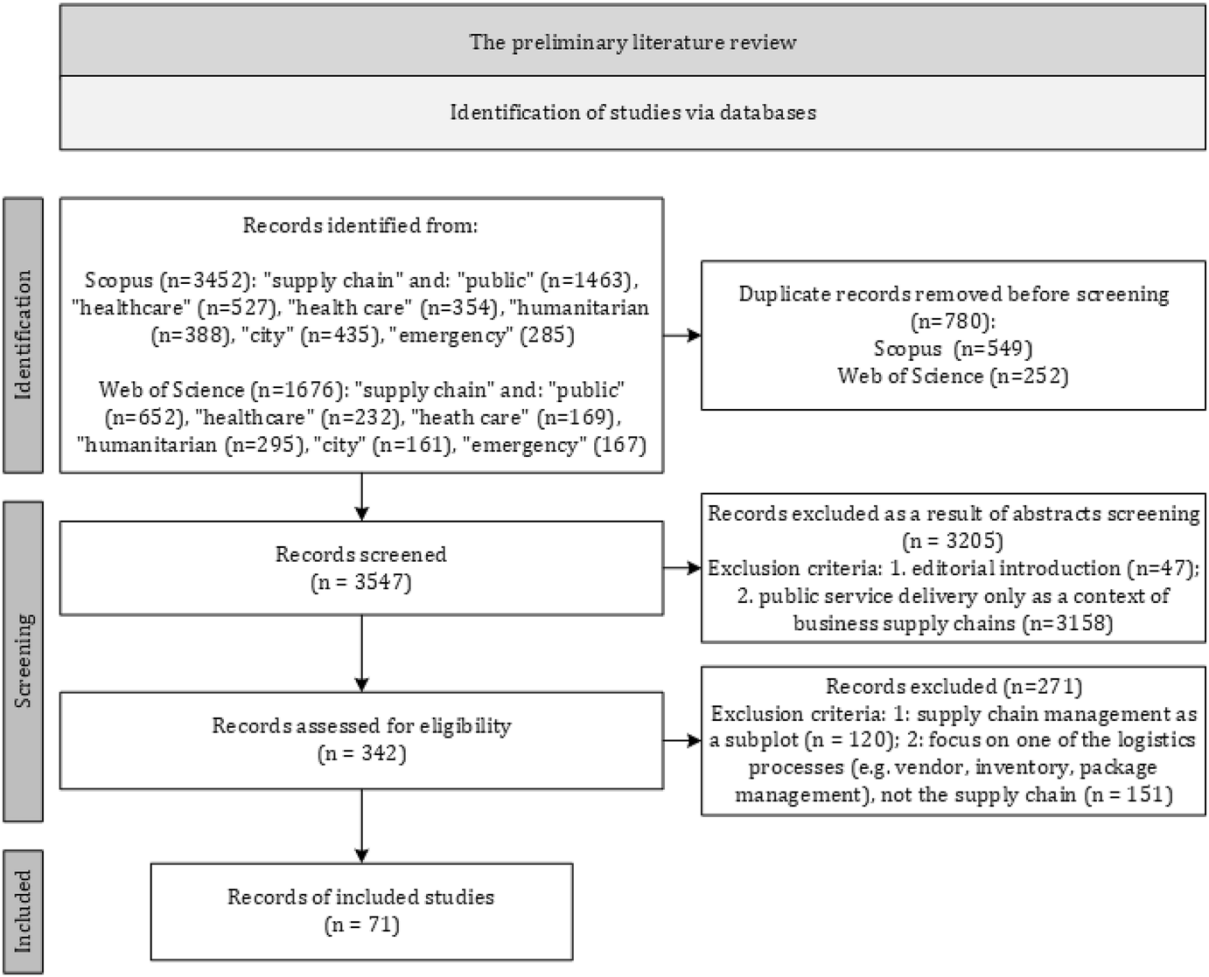
Systematic literature review process diagram. *Source*: Elaboration based on Page et al. (2021)

The search process considers government functions (COFOG 2019) because this is the most general approach that enables identifying the common features of the supply chains that support implementing these functions. COFOG (2019) is the Organization for Economic Co-operation and Development (OECD) and the European Union (EU) standard for the classification of government functions. This paper does not consider the specifics of supply chains (e.g. 3A – agility, adaptability, alignment), their mechanisms (e.g. public–private partnerships or public procurement) and their types (e.g. green supply chains and collaborative supply chains) because they are separate, extensive research areas. The authors intend to systematise the dispersed knowledge on PSSCM in the literature; therefore, they applied a general approach to their search. The first step of the research—identification—was limited to the management and business fields and English-language publications. In total, qualifying records in Scopus totalled 3,452 and in the Web of Science 1,676 records. In the second step, 3,547 records remained after deleting duplicate records. In the third step, within this first phase of screening, the titles, abstracts and keywords were analysed. The criteria for exclusion criteria were: 1. editorial introduction; 2. public service delivery only to contextualise business supply chains. These analyses reduced the number of records to 342. The design of the second phase of screening, using the full text, aimed to identify publications that directly refer to PSSCM issues, using the following exclusion criteria: 1. supply chain management as a subordinate research element; 2. a focus on one of the logistics processes (e.g. vendors, inventory or package management), not the supply chain. The last step identified 71 key publications (Appendix 1). The researchers checked the risk of bias, as Sterne et al. (2019) recommended, and did not code these results, rather using the full records. Following the PRISMA Group framework, Fig. 1 shows the SLR process.

Most excluded records dealt with private-sector-business supply chains, and the public sphere served only for research context. Their titles and abstracts listed the search keywords, but the research context did not apply to public service delivery. This led to the elimination of 3,158 records in the first phase of screening. After thoroughly reading the full text of the remaining publications, the authors identified 120 records that examined public service delivery processes but only mentioned supply chains, without analysing these research issues. Then, 151 publications that related only to logistics processes were also eliminated, to maintain the focus on the supply chain perspective. Thus, the researchers selected the 71 most appropriate publications with which to answer the research questions.

The analysis was based on the study of the content. The intended research scope was also analysed using VOSviewer to visualise the scientific landscapes and PSSCM research issues.

## Research results

### Bibliometric analysis and visualisation of research issues in PSSCM

**Fig. 2:**
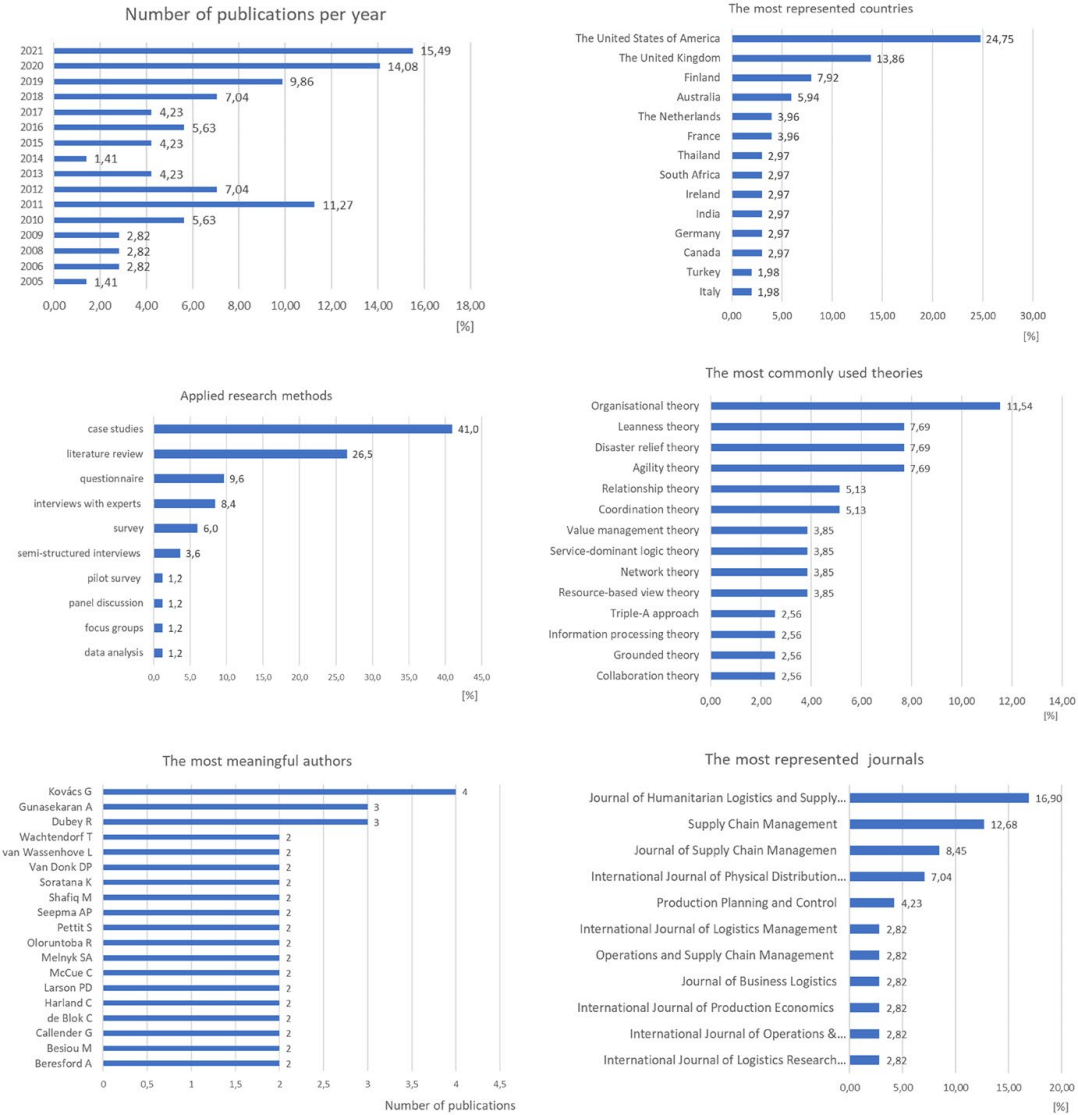
Bibliometric analysis

The bibliometric analysis indicates that supply chain management issues in the public sector have become increasingly popular. Researchers in the United States of America, the United Kingdom, Finland and Australia mainly conduct studies in this area, but studies also occur in other countries worldwide. Case studies and literature reviews are the most popular research methods in identified publications. Besides, theories of organisation, agility, disaster relief and leanness are dominant. The leading journals publishing articles on PSSCM are the *Journal of Humanitarian Logistics and Supply Chain Management*, *Supply Chain Management*, the *Journal of Supply Chain Management* and the *International Journal of Physical Distribution and Logistics Management*. Articles in this research area also, but rarely, appear in journals directly dealing with public sector management, e.g. *Public Money and Management*, the *International Journal of Public Sector Management*, the *American Review of Public Administration* and the *International Public Management Journal*. This proves that although initiatives in this area are manifest mainly among supply chain management researchers, increasingly often, public management researchers see this approach as an opportunity to increase the potential for value creation. A detailed bibliometric analysis appears in Fig. 2 and Appendix 1.

**Fig. 3:**
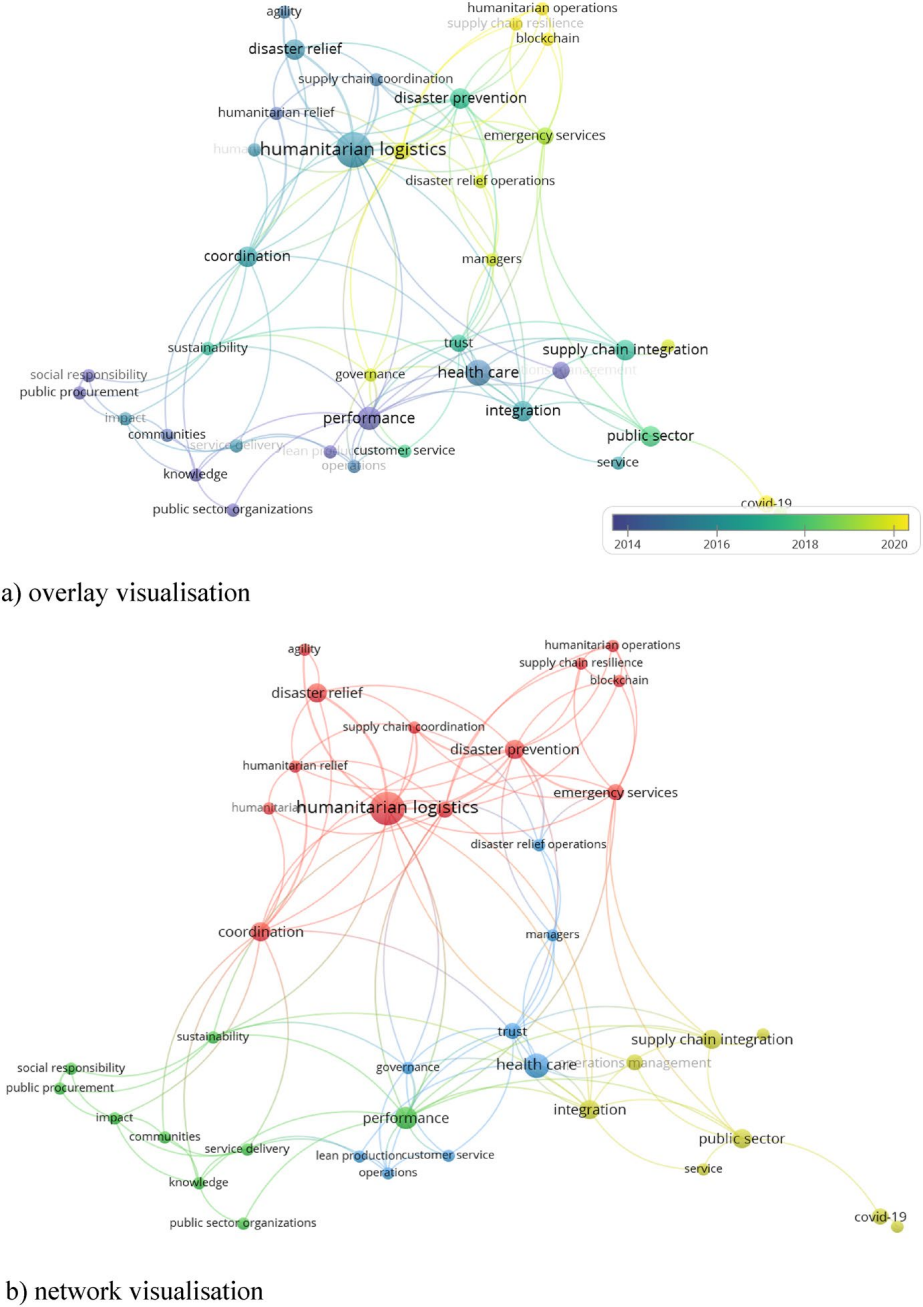
Visualisation of research issues in the PSSCM. *Source*: Elaboration based on the analysis using VOSviewer ver. 1.6.17 as of May 30, 2022

Although the concept of a supply chain in the public sector is not a novelty, most published research on the subject has appeared recently (Fig. 3a). The initial analyses, published until around 2015, aimed to improve the performance of public service delivery processes through public procurement, social responsibility, operations management and leanness. From 2015 to 2018, the research emphasised trust, supply chain integration, agility and supply chain coordination. This research focused on both health care and humanitarian logistics. Nowadays, supply chain research emphasises collaboration, the role of managers, resilience, governance and the use of digital technologies. In addition, the COVID-19 pandemic significantly impacted research development.

The network visualisation of research issues (Fig. 3b) also reflects the evolution of research into PSSCM. As this visualisation shows, the researchers placed traditional research on service delivery performance, public procurement and social responsibility in the first cluster (green). The blue cluster includes studies on health care, including lean production, customer service, disaster relief operations and trust. The red cluster consists of research on humanitarian logistics and emergency management, two research areas very closely thematically related. Their issues relate to traditional research directions (e.g. agility and humanitarian relief) and contemporary trends (e.g. resilience and the blockchain). The yellow cluster includes research that generally relates to the functioning of the public sector, including the integration of supply chains.

### The definition and model of the public service supply chain

The authors of the identified publications note that the need to apply supply chain management (SCM) in the public sector results from the necessity of public service delivery for cross-sectoral collaboration and the need to meet growing social expectations (Gianakis and McCue 2012; Aronsson et al. 2011; Mafini 2016). SCM allows decision-makers to address the problems of functional divisions and smooth the flow of processes. This approach can improve public services delivery by synchronising and integrating resources and coordinating actors’ activities (Akhtar et al. 2012; Radnor and Noke 2013; Aitken et al. 2021; Seepma et al. 2020). The value of public service delivery could be higher. However, the issue of PSSCM involves the implementation of various types of public services (e.g. health care or humanitarian relief) and governance mechanisms (e.g. public–private partnerships or public procurement), primarily occurring at the organisational level (e.g. dyads). Therefore, this paper draws on the public services logic lens and focuses on the most common public services in supply chain literature. According to this perspective, *PSSCM can mean a synchronised process of co-creating value in public administrative networks and ecosystems, in which each actor can function as both a supplier and an end-user, within legal regulations and following the rules of transparency, accountability, equality and publicness. It includes the flows of information, knowledge, activities and tasks between the entities that co-create public services. The needs of society are of key importance, and they determine the scope and manner of providing public services.* The basis of the flows between entities in PSSCM is the fundamental processes of the service supply chain: capacity and skills management, demand management, customer relationship management, supplier relationship management, service delivery management and cash flow management (Ellram et al. 2004). PSSCM does not encompass flows of goods, e.g. the supply of hospital equipment or office supplies. The flow of goods is complementary and supports service delivery in PSSCM. In addition, due to its interorganisational and intersectoral nature and political and social embedding, behavioural factors play a significant role in PSSCM.

Researchers indicate that processes implemented in PSSCM are complex and uncertain and occur under dynamic conditions with limited resources, affecting their performance (Aronsson et al. 2011; Callender 2011; Day et al. 2012; Oloruntoba Kovács 2015; Kunz et al. 2017; Fan et al. 2017; Dolinskaya et al. 2018; Chorfi et al. 2018). These supply chains constitute multidimensional structures of relationships in which actors are autonomous units, with specific management principles and organisational culture that use various technical systems. These actors have different but complementary competencies and are responsible for implementing various processes in the supply chain. Each process proceeds differently, depending on the type of service and the influence of political accountability and public scrutiny (Callender 2011).

**Fig. 4:**
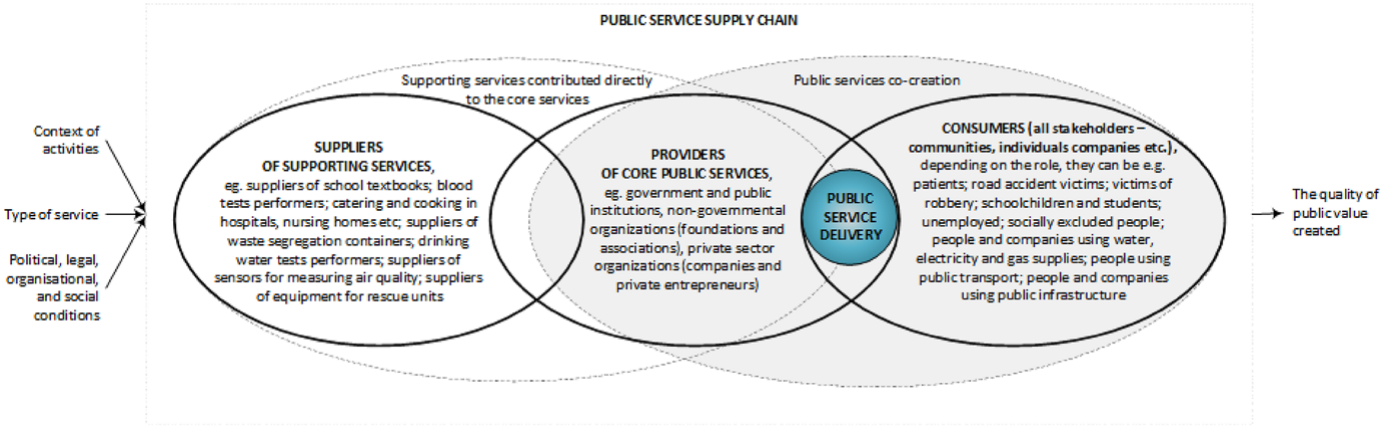
Public service supply chain model. *Source*: Elaboration based on Baltacioglu et al. (2007) and Ellram et al. (2004)

The model that Baltacioglu et al. (2007) developed was derived from the Supply Chain Operation Reference (SCOR) model; the model by Ellram et al. (2004) indicates that the service provider is central to the supply chain that supports the service in creation. The supplier delivers supplementary services to the service provider or the recipient, e.g. the materials necessary to perform the service, maintain relevant infrastructure, conduct laboratory tests or maintain computer applications, including web and mobile applications. Thus, the supplier indirectly contributes to the creation of the service. Service provision occurs when a service provider offers the service to a recipient who consumes it. The recipient also participates in creating the service. Figure 4 shows the model of the supply chain structure and functioning conditions in the public sector.

Based on Baltacioglu et al. (2007), the public service supply chain model in Fig. 4 consists of public service suppliers, providers and customers. Suppliers provide support services for core services. They can provide services directly to the customers if the services are indispensable at the place and time of delivering core services, e.g. nursing services during surgery. Providers directly implement core public services, and customers are all stakeholders who use these services.

Both suppliers and providers can simultaneously be customers of a public service. For example, a person working in a water and sewage company is also a customer of that company, as is a person who tests the quality of the water supply. These three basic units engage in relationships that motivate them to produce the highest quality public services. In addition, customers participate in public service co-creation through (among other things) information about needs, ideas for implementation and resources to design services. The previously described service type, activity context and political, legal, organisational and social conditions also condition the possibilities of the jointly generated value. As part of the management of the public service supply chain, researchers explore many issues. The analyses conducted within the framework of the research presented in this paper identify these issues and divide them into eight major research streams (Appendix 1).

### Research streams in PSSCM

The content analysis of 71 publications was based on a thorough study of the issues they discussed. On this basis, the authors established the main PSSCM research streams (see Appendix 1). By relating them to the work of Lambert and Enz (2017), the division of these publications into research streams appears as follows:

- Structural management components: the structure of PSSCM, interorganisational coordination, cross-sectoral implementation of public tasks, agility-based resilience and digital technologies;
- Behavioural management components: interorganisational collaboration, community-based public service delivery and PSSCM professionals.

The identified research streams complement and influence each other. The structure of PSSCM creates a framework in which public service delivery processes, such as interorganisational coordination and collaboration, operate. Importantly, this structure is cross-sectoral, which is one of the identified research streams. In PSSCM, the public service logic approach highlights two types of actors, public professionals and the community, as those who play a special role in these chains. Moreover, considering the uncertainty and complexity of implementing public services, the research thus far has drawn attention to the concepts of leanness, agility and resilience. Recently, increasing research on the use of digital technologies in PSSCM has appeared. Detailed characteristics of the identified research streams follow.

#### The structure of PSSCM

The structure of PSSCM is one of the primary research directions, due to the complexity and multidimensionality of public services delivery processes. Researchers indicate that the structure of public service supply chains is cross-sectoral, and social needs and legal regulations trigger them (Durugbo et al. 2022; Kebriyaii et al. 2021; Polater and Demirdogen 2018; Lillrank et al. 2011; Balcik et al. 2010). Public agencies play crucial roles in these chains, acting as organisers, facilitators and supply network members (Quarshie and Leuschner 2020; Migiro and Ambe 2008). They have ‘a vast group of stakeholders: those who use the services of the agency and those whose professional work interacts with the agency’ (Callender 2011, p. 14).

Balcik et al. (2010) identify pre- and post-disaster supply flow in the humanitarian supply chain area. The first concerns procurement and stock pre-positioning, and the second addresses procurement and transportation. Dubey et al. (2020, p. 3383) emphasise that ‘the design of humanitarian supply chains must be flexible enough to accommodate the sudden change in demand during disaster relief operations’. Sampson et al. (2015) highlight the need to identify two parallel supply chains in health care. The first supplies equipment and materials, and the second entails service provision. Polater and Demirdogen (2018) also believe that providing complementary flows, such as maintenance and cleaning, always accompanies health services. In every other domain of public governance, auxiliary processes support the provision of public services. Seepma et al. (2021) state that integration mechanisms must support the regulatory structure of the justice supply chain. Accordingly, we conclude that:

##### Proposition 1

Supply chains in the public sector are cross-sectoral and include internal chains that support their external supply chains.

The main topics in this research stream involve the need to build supply chains in the public sector (Polater and Demirdogen 2018; Lillrank et al. 2011), their complexity (Aitken et al. 2021), the differentiation of actors and their roles in these chains (Quarshie and Leuschner 2020; Dobrzykowski 2019) and the distribution of flows (Sampson et al. 2015; Balcik et al. 2010). However, little research investigates integrating internal and external supply chains. Public actors dominate these chains, and opportunities to use the private and nongovernmental sectors remain underdeveloped. Finding research on public supply chain density, transparency or dispersion is also difficult. However, the cross-sectoral implementation of public tasks in PSSCM receives the emphasis.

#### The cross-sectoral implementation of public tasks through public procurement and public–private partnership

Research into PSSCM indicates that two traditional mechanisms regulate private-sector participation in implementing public tasks—public procurement (PP) and public–private partnership (PPP) (Kaupa and Naude 2020; Harland et al. 2019; Amann et al. 2014; Davis and Friske 2013; Stewart et al. 2009). The cross-sectoral implementation of public tasks is a significant research stream in PSSCM because these mechanisms influence supply chain structure and regulate the scope of interoperability between entities in these chains.

Particular attention is paid to sustainable PP. Amann et al. (2014) indicate that public-sector actors achieve more socially responsible goals than environmental goals. Conversely, suppliers handle environmental activities better than socially responsible activities. Brammer and Walker (2011) state that financial factors constitute a key barrier to sustainable PP, which appropriate regulations mainly foster. Researchers underline that improperly conducted PP leads the public to believe that they ‘cannot depend on government to receive the best deal’ (Atkinson et al. 2020, p. 632).

The second widely studied, traditional mechanism of the cross-sectoral implementation of public tasks is a PPP. Davis and Friske (2013) believe that PPP contributes the most to developing hard and soft infrastructure that enables supply chain flows. However, the implementation of PPP entails certain challenges, primarily, the lack of clear guidelines for all partners, difficulties analysing the benefits of new agreements and a focus on results (McAdam et al. 2011). Heaslip and Kovács (2019) suggest using hybrid contracts rather than performance-based agreements to avoid these problems. Moreover, PPP is a mechanism that formally regulates collaboration and, therefore, depends on both public and private sector organisations’ current willingness to collaborate (Davis and Friske 2013). Therefore, we conclude that:

##### Proposition 2

PP and PPP are primary, legitimate formal mechanisms in PSSCM for building cross-sector relationships, which are supposed to be generally available and based on solid regulations to ensure the fairness of operations.

PP and PPP are mechanisms that create the foundations for joint activities. Research on this subject mainly concerns the sustainable delivery of public services (Amann et al. 2014; Brammer and Walker 2011), methods of engaging the private sector (Harland et al. 2019), the difficulties of implementing PP and PPP (McAdam et al. 2011; Davis and Friske 2013) and the effects of these mechanisms (Stewart et al. 2009). Research into the power of PP and PPP to affect PSSCM and the implementation of broader government policies (Harland et al. 2019), the factors of these mechanisms’ performance in PSSCM (Davis and Friske 2013) and building long-term partnerships (McAdam et al. 2011) remains necessary. However, PP and PPP undoubtedly facilitate proactive coordination in supply chains (Stewart et al. 2009).

#### Public service supply chain coordination

The structural and organisational complexity of PSSCM makes coordination crucial for delivering public services and, therefore, is a popular research subject (Durugbo et al. 2022; Okdinawati et al. 2019; Nagurney et al. 2016). Coordination harmonises mutual relations, eliminates problems of shortage or oversupply and maintains a proper flow of information. At the same time, it is a challenging area that requires significant work, time and resources (Seepma et al. 2020; Namagembe 2020; Dobrzykowski 2019; Fu et al. 2006). As a result, many public supply chains struggle to coordinate flows between organisations from different sectors.

Research on coordination in PSSCM mainly relates to its mechanisms, coordinators’ roles and the levels of centralisation and decentralisation (Akhtar et al. 2012; Nagurney et al. 2016; Dobrzykowski 2019). Akhtar et al. (2012, p. 85) found that ‘tangible (finance, technology and people) and intangible (leadership, extra efforts, relevant experiences and education, relationship management skills, research abilities and performance measurement skills) assets are the key determinants of chain‐coordination success’. They also believe that coordination alone does not guarantee success because it does not grant the ability to cope with many interorganisational differences, such as different organisational structures and cultures. Coordinators are expected to adapt effectively to dynamic and unpredictable operating conditions. Therefore, decentralising the supply chain can achieve better results. An example of such a chain is the provision of health care (Hossain et al. 2021; Dobrzykowski 2019). The above findings suggest:

##### Proposition 3

Intersectoral and interorganisational coordination in PSSCM requires decentralisation, continuous structural and cultural adjustment and adaptation to the context and operating conditions.

Researchers emphasise the need to intensify research on coordination in PSSCM, specifically regarding the relationship between coordination and information-sharing policies (Getele et al. 2020), the identification of transparent coordination rules (Ozdemir et al. 2021) and alternative mechanisms (Meijboom et al. 2011), one of which is collaboration (Kunz et al. 2017; Szołtysek and Frączkiewicz-Wronka 2012).

#### Collaboration in PSSCM

The division of competencies of the public organisations responsible for the implementation of specific tasks causes these organisations to combine various relations (e.g. communication, coordination, cooperation and collaboration) (Quarshie and Leuschner 2020). As a result, their activities are interdependent, and they emphasise collaboration that leads to supply chain integration (Balcik et al. 2010; Eckerd and Eckerd 2017).

Many researchers emphasise that developing collaborations in supply chains requires trust (Ozdemir et al. 2021; Chen et al. 2013; Szołtysek and Wronka 2012). Dubey et al. (2019, p. 127–128) believe that a lack of trust is one critical cause of conflict and point to the need for the development of a swift trust that ‘allows individuals to freely interact via information and knowledge sharing, which is often considered critical to the success of collaboration’. Seepma et al. (2020, p. 94) state that based on an analysis of the criminal justice supply chain, ‘laws seem to replace the need for relational integration in building trust and relational norms’. They also note that legal regulations may force collaboration on the one hand and limit contacts on the other. Instead, Eckerd and Eckerd (2017) believe that although formal contract mechanisms dominate the public sector, relational mechanisms operate there, to extents similar to those in the private sector. They also claim that the formal and informal relationship mechanisms in the public sector complement each other. Therefore, legal regulations seem to be a convenient way to regulate the scope of collaboration, especially in situations where trust is low. Oloruntoba and Kovács (2015) share this view, believing that long-term strategic collaboration in humanitarian supply chains is not always optimal. The above findings lead to this proposition:

##### Proposition 4

Within the public sector, legal regulations through which organisations can be controlled and steered condition interorganisational collaboration; nevertheless, this collaboration is more trust-based than intersectoral collaboration, where joint activities are based mostly on contracts and agreements.

Core themes regarding collaboration include the types of collaborative activities (Friday et al. 2021; Eckerd and Eckerd 2017), formal and informal collaboration mechanisms (Chen et al. 2013; Szołtysek and Wronka 2012) and collaboration strategies (Obaze 2019). The need for in-depth research into the causes of collaboration failure and how to strengthen collaboration is also emphasised (Dubey and Gunasekaran 2016; McKone-Sweet et al. 2005). Furthermore, the role of society is significant in the field of collaboration (Pillitteri et al. 2021; Obaze 2019).

#### Community-based PSSCM

Researchers devote a significant part of the research into PSSCM to the co-production and co-creation of public value. This orientation results from how supply chain management favours the creation of appropriate structures and strategies to satisfy the end customer (Pillitteri et al. 2021; Obaze 2019). From this perspective, managing relationships within supply chains to support the development of interactions with local communities and the possibility of exchanging services for services is beneficial. It encourages the beneficiaries to adapt to changes and learn and develop coping skills. According to Dobrzykowski (2019), the need to involve beneficiaries in value-creation processes is apparent in health care, where information is constantly shared between the patient and the doctor for diagnosis and treatment. Experience in ​​humanitarian logistics shows that co-production improves the identification of needs and the development of the local economy (Kovács et al. 2010). Therefore, greater community involvement in providing public service allows the transparency and transformation of these processes to increase, better serving beneficiaries’ needs (Obaze 2019; Kovács et al. 2010).

Obaze (2019, p. 419) emphasises that ‘services become transformational when embedded into systems, promote relational benefits, service design, service practices and social structures’. Contextual knowledge from consultations, social participation and the development of relational and communication skills with local communities fosters this embedding (Pillitteri et al. 2021; Kovács et al. 2010). In addition, designing supply chains around them can facilitate beneficiaries’ active role (Kovács et al. 2010). Therefore, the authors made another assumption:

##### Proposition 5

PSSCM embeddedness among beneficiaries increases the co-creation of public value, transparency and the transformative nature of public service delivery processes.

This research stream requires a significant intensification of research. So far, limited research on this topic has been devoted primarily to the benefits and challenges of implementing a community-based approach (Kovács et al. 2010). This study recommends directing more attention to the social dimensions of supply chains in the public sector (Dubey and Gunasekaran 2016), interactions within community-based settings (Obaze 2019) and a ‘dynamic view of supply chain design’ (Kovács et al. 2010, p. 421). In addition, engaging the right professionals is essential for supporting community-oriented supply chains in the public sector (Pillitteri et al. 2021; Atkinson et al. 2020).

#### PSSCM professionals

PSSCM professionals fulfil many functions, including serving as coordinators, integrators, facilitators and leaders (Pillitteri et al. 2021; Atkinson et al. 2020; Gianakis and McCue 2012; Akhtar et al. 2012). However, their primary role in PSSCM is to develop and maintain relationships with beneficiaries to co-create public value (Pillitteri et al. 2021). Achieving this requires professionals to persuade and guide beneficiaries beyond simply involving them in public service delivery processes based on standard operating procedures (Pillitteri et al. 2021).

Professionals implementing tasks in the public sector is challenging. Gianakis and McCue (2012) state that accountability increases public professionals’ risk aversion beyond what private-sector professionals experience. According to Atkinson et al. (2020), political conditions may drive professionals to surround themselves with flatterers rather than experts. In addition, differences in culture and organisational structure, financial constraints or coordination problems can significantly limit interorganisational relationships and the co-creation of public value (Akhtar et al. 2012). This situational complexity requires PSSCM professionals to have high-level social competencies, particularly in soft skills. Pillitteri et al. (2021) emphasise that trust-based relationships for value co-creation can develop based on professionals’ knowledge, beneficiaries’ orientation and effective communication. McKone-Sweet et al. (2005) and Getele et al. (2020) also indicate the need for professionals with social competencies, including communication skills and the ability to collaborate. These findings lead to this conclusion:

##### Proposition 6

Public professionals with high-level social competencies who are focused on developing interorganisational and intersectoral relationships and motivating beneficiaries to participate in public service delivery processes are necessary to co-create public value in PSSCM.

The changing role of professionals in PSSCM is a significant research challenge (Pillitteri et al. 2021; Atkinson et al. 2020). They are no longer only public managers but also public servants who work interorganisationally and cross-sectorally to provide the highest quality public services. Previous research on this topic emphasises PSSCM professionals’ roles (e.g. McCue 2012; Akhtar et al. 2012), competencies (McKone-Sweet et al. 2005) and action motivators (e.g. Gianakis and McCue 2012). However, this research topic is still in its infancy, and the need persists for in-depth research on highly skilled professionals (Pillitteri et al. 2021; Getele et al. 2020). Such actors can significantly affect supply chains’ organisational capacity to prevent and/or respond to dynamically changing contexts of operation.

#### The leagility-based resilience of PSSCM

Recent studies on PSSC functioning indicate the need to develop the resilience of these chains (Scala and Lindsay 2021; Friday et al. 2021; Atkinson et al. 2020; Shafiq and Soratana 2020; Oloruntoba and Kovács 2015; Stewart et al. 2009). This need for resilience results from the societal consequences of disrupting supply chains. People worldwide experienced the multiple effects of interrupted supply chains with the Covid-19 pandemic that began in 2020 and Russia’s aggression towards Ukraine in 2022. Both the pandemic and the war have shown weaknesses in PSSCM. Supply chain breakage has painful effects on public services in light of its association with the exposure of society to danger. Research on this topic highlights the importance of collaboration, flexibility, leanness and agility in building resilient supply chains (Friday et al. 2021; Dixit et al. 2021; Scala and Lindsay 2021; Nayak and Choudhary 2020; Shafiq and Soratana 2019; Pettit et al. 2016; Dubey and Gunasekaran 2016; Hines et al. 2008).

Hines et al. (2008) show that implementing leanness in public services requires more emphasis than in the private sector on the social aspect. This concept’s association with agility arises often, in its capacity as ‘the ability to respond quickly and adequately to short-term changes in demand, supply or the environment’ (Charles et al. 2010, p. 727). Agility in public services literature refers to identifying and responding to real demand (Nelan et al. 2018; Stentoft Arlbjørn et al. 2011; Oloruntoba and Gray 2006). However, as L’Hermitte et al. (2015) emphasise, agility is about not only flexible supply positioning but also understanding problems and changing situational conditions, striving for a balance between formal procedures and decentralised initiatives and developing strategies to address challenges. Agility and leanness allow professionals to ensure an efficient flow of public service delivery processes.

Nayak and Choudhary (2020) state that a leagile strategy is beneficial in humanitarian SCM. In their proposal, leanness is applied upstream, before the decoupling point, and agility can be applied downstream in the supply chain. Scholten et al. (2010) come to similar conclusions, but they also believe that implementing leagility can allow the parallel management of different supply chains. Meanwhile, Aronsson et al. (2011) concluded that health care supply chains need a greater focus on agility due to the diversity of their reaction and execution times. These findings indicate that the appropriate combination of both concepts in the relevant parts of the supply chain is crucial. Falagara Sigala et al. (2020) also believe that, along with agility, professionals must consider adaptability and alignment (Triple-A). These authors prove that Triple-A enables a rapid response to unexpected changes in operating conditions and the coordination of interorganisational processes. The above considerations lead to this conclusion:

##### Proposition 7

The merger of agility and leanness to adapt to a specific context of operation creates foundations for ensuring the resilience of PSSCM.

Leanness, agility and resilience in business supply chains are widely studied. In the public sector, such research remains scattered and focuses mainly on explaining these concepts’ roles in supply chains (Nayak and Choudhary 2020), their effects (Nelan et al. 2018; Stentoft Arlbjørn et al. 2011), driving factors (L’Hermitte et al. 2015) and the possibility of combining them with other strategies (Falagara Sigala et al. 2020). According to Nelan et al. (2018), Aronsson et al. (2011) and Hines et al. (2008), research is needed on developing leanness, agility and resilience in PSSCM, especially in complex and uncertain circumstances. The conditions for developing such organisational abilities can create digital technologies.

#### Digital technologies in PSSCM

The dynamic technological progress that has been visible in recent years has revealed new opportunities for public professionals to improve the processes of public service delivery (Durugbo et al. 2022; Nelan et al. 2018; Pettit et al. 2016; Chen et al. 2013; Gianakis and McCue 2012). Good-quality data allows for an efficient process and encourages relationship management, not firefighting (Falagara Sigala et al. 2020, p. 232).

The implementation of digital technologies is likely to significantly affect PSSCM. Digital technologies could improve intra- and interorganisational communication, streamline decision-making processes and expand the Triple-A dimensions of supply chains (Falagara Sigala et al. 2020). One of the most frequently mentioned technologies in PSSCM is information and communication technologies (ICT). When adequately managed, they can increase supply chain efficiency and transparency and improve accountability (Dixit et al. 2021; Seepma et al. 2020; Pettit et al. 2016; Kritchanchai 2012; Okdinawati et al. 2019).

Recently, blockchain technology has received considerable attention due to supply chains’ increasing complexity and their interorganisational nature. The blockchain enables secure transactions through a decentralised ledger that users can access without the possibility of retroactive changes (Ozdemir et al. 2021; Dubey et al. 2020). This technology can help to build transparent relationships in the supply chain, improve interorganisational coordination and ensure flow control. The World Food Programme and The International Federation of Red Cross and Red Crescent Societies already use the blockchain (Ozdemir et al. 2021).

However, Seepma et al. (2020) emphasise that the use of digital technologies does not automatically increase efficiency in SCM. It requires the simultaneous implementation of organisational and management changes and contextual adjustment. Digital technology is an enabling tool. The above findings lead to this conclusion:

##### Proposition 8

Digital technologies are tools that can help public professionals to address problems in communication and coordination throughout PSSCM, but they require appropriate management, awareness of their advantages and limitations and adaptation to the realised processes and context of public service delivery*.*

Previous studies on the use of digital technologies in public sector supply chains refer to their effects (Dixit et al. 2021; Falagara Sigala et al. 2020) and the conditions for implementing and utilising them (Seepma et al. 2020). However, to indicate (a) what barriers to digital technology usage may appear and how to overcome these barriers (Dubey et al. 2020), (b) how to integrate the use of various technologies in supply chains (Falagara Sigala et al. 2020) and (c) the attitude of different users towards providing public services digitally, requires more empirical research and case studies (Seepma et al. 2021).

## Discussion

PSSCM is becoming an increasingly popular and desirable research area. The analyses on this topic indicate that this approach increases the effectiveness of the actions taken (Atkinson et al. 2020; Mafini 2016; McLachlin and Larson 2011; Aronsson et al. 2011). Both supply chain and public governance researchers share this view. However, the relative scarcity of analyses undertaken from the PSSCM perspective (Harland et al. 2019) and the dispersion of previous research across various areas of public governance (COFOG 2019) leave PSSCM underdeveloped (Mafini 2016). By merging knowledge from the most-investigated areas of public governance in the SCM field, this paper attempts to close that research gap.

This paper defines PSSCM as a synchronised process of co-creating value in public administrative networks and ecosystems, during which each actor can be both a supplier and an end-user, within legal regulations and the rules of transparency, accountability, equality and publicness. It includes the flows of information, knowledge, activities and tasks that must occur among the entities co-creating public service. Societal needs are of key importance; they determine the scope and manner of providing public services. The foundation of this definition is a general understanding of the service supply chain as a network of actors performing different transactional functions, aiming to configure services that meet the expectations of their beneficiaries, as Arlbjørn et al. (2011) and Baltacioglu et al. (2007) emphasise. It refers to the intangible values developed through interorganisational and cross-sector interactions, in which the flows of goods support service configurations. This definition also incorporates the crucial role of society’s needs when determining the types of public services and the means of offering them, considering the rules of process management in these administrative networks and public value co-creation, as Osborne (2021), Petrescu (2019) and Ellram et al. (2004) emphasise. Therefore, it combines the basics of service SCM with public value and public service logic theories.

The public service supply chain structure (Fig. 4) is much shorter than that of traditional goods supply chains. In the public service supply chain, the supplier network is less extensive than, for example, in the SCOR model, but it only applies to suppliers that support public service co-creation. Similarly, the client and the customer can be one actor because of the simultaneity of services and their implementation in the presence of both the service provider and the recipient. The model that appears in Fig. 4 is an adapted version of the models of Baltacioglu et al. (2007) and Ellram et al. (2004) for the processes of delivering public services from the public service logic perspective. The resulting model highlights such characteristics of the service supply chain as the simultaneous creation and consumption of services, the possibility for actors to be both service providers and recipients and the heterogeneity of services, which stakeholders perceive differently with every use. This model also indicates that public value co-creation occurs during public service use and consumption, which aligns with public service logic theory (Petrescu 2019; Osborne 2021). It emphasises the specificity of PSSCM, resulting from the context of operation, political and organisational conditions and a wide range of public services. Moreover, this specificity reveals the impossibility of public services adopting private-sector supply chain practices; implementing supply chain thinking in the public sector is preferable.

Analysis of the research on the evolution of PSSCM indicates that the early development of this research area can be associated with PP, social responsibility and lean practices implementation aiming to achieve higher levels of performance in public organisations. These are the issues underlying the development of SCM in the public sector and the creation of mechanisms for developing and structuring relationships between organisations from various sectors (Karaba et al. 2022; Piotrowicz et al. 2022; Howard et al. 2017; Zheng et al. 2008). These mechanisms have formal natures, and relational elements supplement them in the later research (Aben et al. 2021; Caldwell et al. 2017; Lambert and Enz 2017). Therefore, the latest research focuses on reinforcing collaboration and leagility to build organisational resilience (Sienkiewicz-Małyjurek 2022; Scala and Lindsay 2021; Friday et al. 2021; Ozdemir et al. 2021; Chen et al. 2013), in answer to contemporary governance problems of uncertainty and the growing scale of supply chain threats (Phillips et al. 2022; Sienkiewicz-Małyjurek 2022). This study also shows that the dynamically growing digitisation of public services constitutes a prospective research area that offers the potential for many quality improvements to public service delivery processes. For example, Durugbo et al. (2022), Aben et al. (2021) and Szymczak (2019) emphasised the research needs in this area. Moreover, the analysis of the relationship between research issues in PSSCM shows strong connections, pointing to the legitimacy of the holistic approach this paper adopted.

A significant finding of this paper is the identification of eight major research streams in PSSCM, streams that are common to all chosen research areas. Only the context of an activity affects the different approaches to its practical application. For example, humanitarian relief emphasises the importance of the identified research streams for tracking material and material donations, involving nongovernmental organisations and volunteers in activities and coping with dynamic and uncertain conditions (Ozdemir et al. 2021; Obaze 2019; Nelan et al. 2018). In health care, integrating internal and external supply chains (e.g. the supply of drugs and hygiene products adapted to treatment processes) reflects these research streams. They are also helpful in ensuring the appropriate auxiliary services (e.g. catering, washing and cleaning) and value co-creation (Aronsson et al. 2011). Most likely, other PSSCM areas require investigation and analysis, e.g. sustainability, risk or security. In addition, the areas this paper identifies are extensive research issues that become the various lenses through which to analyse PSSCM, e.g. collaboration and coordination failures (Kalra et al. 2021), the drivers of digital technologies (Aben et al. 2021), mechanisms of leanness and agility (Piotrowicz et al. 2022), the role of contracts in cross-sector collaboration (Karaba et al. 2022), managing information asymmetry (Phillips et al. 2021) or collaborative trust (Kożuch and Sienkiewicz-Małyjurek 2022).

By analysing the results of the recent research on criminal justice supply chains, Seepma et al. (2020) found that interorganisational relations in the public sector are primarily contractual and regulatory. They believe that relational integration is almost absent. Similarly, Marques et al. (2020), during research into the use of technology in health care, found a lack of research investigating collaboration. Meanwhile, Quarshie and Leuschner (2020) confirmed the presence, importance and extensive research on the 4Cs (communication, coordination, cooperation and collaboration) of emergency management. Research on collaboration in the humanitarian supply chain also confirms the implementation of operations intended to strengthen interorganisational collaboration (Dubey et al. 2020; McLachlin and Larson 2011). In addition, for many years, the literature on public governance has presented the results of intensive research on public networks and collaborative governance (Klijn and Koppenjan 2016; Emerson et al. 2012; Ansell and Gash 2008). For this topic, the development of intersectoral and interorganisational relations is crucial. Although building interorganisational collaboration in the public sector requires more time and caution than doing so in the private sector, these relations exist and are in development. The basis of public service logic lies in conducting activities within networks where each actor can simultaneously play different roles to co-create value based on mutual trust (Kożuch and Sienkiewicz-Małyjurek 2022; Osborne 2021; Engen et al. 2020; Petrescu 2019). According to Aben et al. (2021), relational governance is the best way to solve problems in PPP. Although the relationships may differ in nature and intensity between public and private supply chains, collaboration in the public sector is recognised as a powerful mechanism of service delivery (Scala and Lindsay 2021; Dubey et al. 2019; Meijboom et al. 2011; McLachlin and Larson 2011). Therefore, significant discrepancies arise in considering the role and level of interorganisational relations in PSSCM. Moreover, supply chain integration processes far surpass typical organisational relationships. Within public services, the recipients are susceptible to the value they derive from service providers, seeing it as a result of spending public money gathered via taxes. A positive assessment of the value public services provide, resulting from citizens’ translating the consumption of these services into their well-being, further translates into supporting the authorities who organise the processes of public services delivery. According to Meijboom et al. (2011) and Polater and Demirdogen (2018), complete internal and external integration is necessary to meet health care requirements. Many authors believe that such integration should be based on collaborative forecasting and demand planning, replenishment and risk management (Friday et al. 2021; Polater and Demirdogen 2018). Alternatively, Seepma et al. (2020) argue that criminal justice integration does not have to be complete, as a high level of supply chain integration is not always desirable. However, the level of integration should be adapted to each field’s type of relationship. This diversity of views emphasises that the implementation of each operation requires an individual approach, and the role of context in shaping supply chains in the public sector is relevant.

Consequently, this paper posits the difference between considering public service delivery in the context of SCM and from public service logic perspectives. The perspectives develop in parallel, rarely intersecting, which contributes to the emergence of discrepancies that may lead to conflicting findings and practical recommendations. The results this paper presents highlight a need to combine perspectives because of their orientation towards the service-dominant logic approach, based on cross-sectoral and interorganisational collaboration and aiming to include final customers (in this case, citizens and communities) in creating public value and providing public services. This combination may affect finding opportunities to increase the quality of public service delivery through SCM thinking within the public service logic paradigm.

## Conclusions

This study sought to better understand SCM in public service delivery processes. It used an SLR methodology to analyse and combine achievements in PSSCM from the perspective of implementing various government functions. The findings offer theoretical and managerial contributions, as well as some limitations.

### Theoretical implications

The study results contribute to the existing literature in two respects.

First, this paper adds value to supply chain management research by organising its achievements in public service delivery, defining PSSCM and building a model of the public service supply chain. Unlike the extant research that mainly focuses on specific government functions (e.g. health care, emergency management, humanitarian relief, city management), this paper adopts a comprehensive perspective on PSSCM. Although researchers see potential in this direction of study, it remains underdeveloped (Mafini 2016; Dobrzykowski 2019; Seepma et al. 2020). Similarly, public service logic theory can take advantage of the supply chain concept through its orientation towards identifying information and knowledge flows between actors, analysing the course of transforming resources into public services and monitoring the methods of service provision. Thus, this paper presents new research perspectives that may increase the quality of public services and add value to the service-dominant logic perspective.

Second, these research findings contribute to a better understanding of SCM in public service delivery processes, by identifying research streams in this area. Previous studies indicate that the delivery of public services is cross-sectoral and involves many organisations whose activities are interdependent and occur under dynamic, uncertain conditions (Fan et al. 2017; Oloruntoba and Kovács 2015; Aronsson et al. 2011; Callender 2011). Simultaneously, resource constraints and weaknesses in transferring processes between the actors in the chain occur (Dolinskaya et al. 2018; Meijboom et al. 2011; Aronsson et al. 2011). The paper reveals that these factors require a decentralised, context-based and leagile approach to public service delivery. Fulfilling these requirements could build the PSSCM’s resilience and adapt public service delivery to the needs of local users. In particular, leagility may facilitate coordinating internal and external supply chains and adjusting the integration level of these chains according to the operational context. Moreover, the findings here highlight the key role of beneficiaries in value co-creation; public professionals should design public service supply chains around them. These professionals should also strive to develop interorganisational and intersectoral collaboration and motivate beneficiaries to co-create public value. Technological solutions can assist in this purpose, although their implementation alone is insufficient; they require management for best results. Altogether, these issues can enhance the quality of public service delivery processes.

As a result, this paper offers new insight through a holistic view of SCM in public service delivery. Thus, it meets the expectations of Harland et al. (2019), Chorfi et al. (2018), Mafini (2016) and Fan et al. (2017), identifying the current achievements in the implementation of SCM in the public sector as well as future opportunities and needs in this area.

### Managerial implications

This study has important implications for managers in the public sector seeking ways to increase the quality of public services. It emphasises the need for a holistic approach to PSSCM that considers the diversity and specificity of public services, the cross-sectoral need to implement public tasks and the uncertain and dynamic circumstances of realised processes. By adopting and implementing supply chain thinking, public managers could achieve higher-quality public services.

Therefore, public managers should treat such formal mechanisms as public procurement, contracts and public–private partnerships as customary because they are the foundation of cross-sectoral collaboration. However, achieving higher-quality public services also requires developing relational mechanisms and collaboration skills. Relational mechanisms, such as trust, improve the understanding of other actors’ expectations in the cross-sectoral implementation of public tasks and increase involvement in joint activities beyond that specified in agreements and contracts. Public managers should also adjust coordination to the service context and operating conditions, as PSSCM differs in each case. As the literature highlights, problems of coordination failure require an approach that is appropriate for a given supply chain. In addition, merging agility and leanness can ensure the responsible utilisation of resources as well as a quick response to changing operating conditions and customer requirements. PSSCM managers should invest in compatible digital technologies to improve communication and interorganisational coordination. Hence, this study provides levers for public managers to use to enhance the quality of public service delivery processes.

This study encourages public managers to implement and use supply chain thinking in public service delivery processes. It reveals that supply chain thinking could be a good way to increase public governance performance and the quality of public services. The supply chain core is optimisation, cost reduction and efficiency gains in interorganisational activities to meet customer needs (Aronsson et al. 2011; Chopra and Meindl 2004). This approach is also a key goal of public governance, which seeks value co-creation to meet the needs of each member of society. Therefore, public managers can take advantage of the eight research streams this paper identified and develop them in their supply chains.

When building public service delivery networks, professionals also must understand the interdisciplinary nature of these processes. Therefore, when planning the scope and course of public service provision processes, they should build interdisciplinary teams that include public policy experts and experts in SCM. These teams should include IT specialists who support the digital side of supply chains. This research also indicates that service co-creation and citizen involvement are fundamental to PSSCM. Therefore, managers should view citizens as their main partners in planning and delivering public services.

To sum up, this article's practical implications emphasise that the choice of how to provide public services should result from the type of relevant service and the context of activities, together with political, legal, organisational and social conditions. These implications also indicate the need to involve entities from various sectors as well as beneficiaries of these services in the entire public service delivery process. In addition, the appropriate use of digital technologies can foster communication and coordination in providing public services. Supply chain thinking should be embedded in public services management practice (as PSSCM imposes it), underpinned by a system-wide interdisciplinary approach and boosted by cutting-edge information and communications technology.

### Limitations and future research directions

The research is not free from limitations. First, only the areas of public governance present most frequently in SCM were included in the analyses. These were general public services, health care, humanitarian relief, emergency management and city management. SCM may have also emerged in other government functions, e.g. social care and education, and future research could verify this.

Second, the research in this paper is based only on the analysis of public service delivery processes from the perspective of SCM. The operational approach was omitted, and no link between government functions and logistics was sought. This limitation results from the specificity of public service logic, which defines public service delivery as occurring in networks and service ecosystems where actors collaborate and co-create value. However, including an operational approach and using ‘logistics’ in the search process could enrich the results of this study.

These research limitations generate interesting and promising follow-up research opportunities. There is a primary need for deeper research into PSSCM, especially from an interdisciplinary perspective. Future analyses could consider other government functions than those the study mentioned. Some suggestions for future research may include:

- case studies of the implementation of individual public services from the perspective of PSSCM, e.g. care for the elderly, implementation of preventive operations by the police, energy supply, waste management, wastewater treatment, provision of cultural services;
- analysis of the impact of PSSCM on the performance of public service delivery processes;
- identifying drivers and constraints of PSSCM in public service delivery processes;
- analysis of factors affecting the actions of public service actors in the relevant supply chain model;
- presenting and characterising how to use digital technologies to improve public service delivery processes from the PSSCM perspective and investigating the impact of selected technologies on PSSCM efficiency;
- presenting a proposal for a comprehensive, PSSCM-based public governance system and distinguishing the most important relationships between its elements, based on evaluating them from the perspective of creating and delivering value to all stakeholders and all groups of citizens;
- checking the operational approach to public services delivery by considering the scope of the term ‘logistics’ in the literature on these services, delivering and comparing the results obtained with the propositions of this article.

Moreover, the propositions of this paper should be empirically verified by analysing a range of case studies on the implementation of various government functions, comparing them and formulating generalisations from them. In this paper, we only present the tip of the iceberg and hope to support significant further theoretical and empirical research.

## Data Availability

The authors declare that data generated and analysed during this study are included in this paper and summarised in Appendix 1. Any additional clarification is available from the corresponding author.

## Appendix 1

**Table:**

Authors	Source	Year	Purpose	Country	Method	Theory/lens used	Scope	Research stream							
								Structure of the PSSCM	Relationship management in the PSSCM	Public service supply chain coordination	Cross-sectoral implementation of public tasks	Community-based PSSCM	Professionals of the PSSCM	Leagility-based resilience of PSSCM	Digital technologies in the PSSCM
Aitken J, Esain AE, Williams S	Supply Chain Management	2021	Examining the sources, types and nature of complexity within a care ecosystem in the UK	United Kingdom	Case study	Service-dominant logic theory	Healthcare	x	x			x			
Akhtar P, Marr NE, Garnevska EV	Journal of Humanitarian Logistics and Supply Chain Management	2012	To identify chain coordinators and explore their roles	New Zealand	Case study	Coordination theory	Humanitarian aid	x		x			x		
Amann MK, Roehrich J, Eßig M, Harland C	Supply Chain Management	2014	To provide evidence of connections between sustainability policy goals included in public procurement tenders and offers and their achievement through contract awards	Germany; United Kingdom	Survey	Inducement–contribution theory	Public services generally		x		x				
Arlbjørn JS, Vagn Freytag P, de Haas H	International Journal of Physical Distribution & Logistics Management	2011	To investigate lean practices in the municipal sector in a service supply chain management (SCM) context	Denmark	Questionnaire	Lean management	Public services generally		x					x	
Aronsson H, Abrahamsson M, Spens K	Supply Chain Management	2011	To find out what is important to consider when developing a supply chain in health care, what is required in order to establish a supply chain orientation and how lean and agile can be used as process strategies in order to improve supply chain performance	Sweden, Finland	Case studies	Agility and leanness theories	Healthcare	x		x				x	
Atkinson CL, McCue C, Prier E, Atkinson AM	American Review of Public Administration	2020	Identification of documentable public failures that provide a deeper understanding of the US government COVID-19 responses’ impact on supply chains	United States of America	Case studies	Disaster management theory	Public services generally		x		x		x		
Balcik B, Beamon BM, Krejci CC, Muramatsu KM, Ramirez M	International Journal of Production Economics	2010	To review the challenges in coordinating humanitarian relief chains, describe the current and emerging coordination practices in disaster relief, examine some widely practised supply chain coordination mechanisms and evaluate their adaptability to the unique relief environment	United States of America	Literature review	Coordination theory	Humanitarian aid	x	x	x	x				
Brammer S, Walker H	International Journal of Operations & Production Management	2011	To identify how international public sector organisations respond to this encouragement or of the conditions most conducive to sustainable procurement	United Kingdom	Survey	Sustainability theory	Public services generally				x		x		
Callender G	International Journal of Productivity and Performance Management	2011	To explore sources of political and administrative challenges which arise from an absence of alignment of supply chains linking the activities of public agencies	Australia	Literature review, case study	Alignment theory	Public services generally	x	x				x		
Chakraborty S, Gonzalez JA	Operations and Supply Chain Management	2018	To offer an integrated supply chain perspective of the US hospital ecosystem that suggests ways for hospitals to operate more efficiently, reduce costs, and improve care quality	United States of America	Literature review	Lean management	Healthcare	x	x						x
Charles A, Lauras M, van Wassenhove L	International Journal of Physical Distribution and Logistics Management	2010	To clearly define the concept of supply chain agility and to build a model for assessing the level of agility of a supply chain	France	Literature review, case study	Agility theory	Humanitarian supply chain							x	
Chen DQ, Preston DS, Xia W	Journal of Operations Management	2013	To propose a research model based on a relational view, delineating the factors that influence hospital supply chain performance: trust, knowledge exchange, IT integration between the hospital and its suppliers, and hospital–supplier integration	United States of America	Survey	Relational view of competitive advantage	Healthcare		x						x
Chorfi Z, Benabbou L, Berrado A	Supply Chain Forum	2018	To engineer an integrated performance measurement system for managing public healthcare supply chains	Morocco	Literature review, case study	Performance management	Healthcare	x			x				
Davis DF, Friske W	Journal of Business Logistics	2013	To develop empirically based theoretical insights into the nature and role of PPPs in the context of cross-border logistics	United States of America	Case study	Grounded theory	Public services generally		x		x				
Day JM, Melnyk SA, Larson PD, Davis EW, Whybark DC	Journal of Supply Chain Management	2012	To position humanitarian and disaster relief supply chains (HDRSC) within the broad field of supply chain management	United States of America, Canada	Interview with experts	Disaster relief	Humanitarian aid			x					x
Dixit A, Routroy S, Dubey SK	International Journal of Quality & Reliability Management	2021	Implementation of supply chain value stream mapping (SCVSM) in a government-supported drug distribution system (DDS) for enhanced patient satisfaction	India	Case study	Value management	Healthcare							x	x
Dobrzykowski D	Journal of Supply Chain Management	2019	To understand how key government regulations affect coordination in the downstream healthcare delivery supply chain	United States of America	Literature review	Institutional theory	Healthcare	x	x	x		x			x
Dolinskaya I, Besiou M, Guerrero-Garcia S	Journal of Humanitarian Logistics and Supply Chain Management	2018	To discuss large-scale disaster and medical assistance as a critical component of the emergency response	United States of America, Germany	Literature review, interviews with experts, case study	Disaster relief	Humanitarian aid	x		x	x				
Dubey R, Gunasekaran A	International Journal of Logistics Research and Applications	2016	To provide a sustainable humanitarian supply chain (SHSC) definition, propose an SHSC structure and validate it empirically	India, United States of America	Literature review, consulting experts, pilot survey	Triple-A	Humanitarian aid	x	x					x	
Dubey R, Gunasekaran A, Bryde DJ, Dwivedi YK, Papadopoulos T	International Journal of Production Research	2020	To understand how big data analytics capability (BDAC) as an organisational culture can enhance trust and collaborative performance between civil and military organisations engaged in disaster relief operations	France, United States of America, United Kingdom	Questionnaire	Organisational information processing theory	Humanitarian aid		x						x
Dubey R, Gunasekaran A, Childe SJ, Roubaud D, Fosso Wamba S, Giannakis M, Foropon C	International Journal of Production Economics	2019	Proposing a theoretical model to understand how blockchain technology (BT) can influence operational supply chain transparency (OSTC) and swift trust (ST)	France, United States of America, United Kingdom	Questionnaire	Organisational information processing theory	Humanitarian aid	x	x						x
Durugbo CM, Almahamid SM, Budalamah LH, Al-Jayyousi OR, BendiMerad B	Journal of Humanitarian Logistics and Supply Chain Management	2021	To explore regional logistics management in times of crisis from the perspective of public authorities responsible for combating the COVID-19 pandemic	Bahrain	Case study	Organisation theory	Humanitarian aid	x	x	x				x	x
Eckerd A, Eckerd S	International Public Management Journal	2017	Comparing the governance mechanisms that public sector managers and private sector managers leverage when responding to conflict in supply chain relationships	United States of America	Survey	Relationships management	Public services generally		x				x		
Falagara Sigala I, Kettinger WJ, Wakolbinger T	Journal of Humanitarian Logistics and Supply Chain Management	2020	To explore what design principles need to be considered in Enterprise Resource Planning (ERP) systems for humanitarian organisations (Hos) to enable agile, adaptive and aligned (Triple-A) humanitarian supply chain capabilities and digitise humanitarian operations	Austria, United States of America	Case study	Triple-A	Humanitarian aid		x					x	x
Fan Y, French ML, Duray R, Stading GL	Service Industries Journal	2017	To determine the effect of environmental uncertainty and strategic choices on operational capabilities in emergency services under different governance structures	United States of America	Data analysis	Environmental uncertainty and the strategic design choices	Public services generally		x	x	x		x		
Friday D, Savage DA, Melnyk SA, Harrison N, Ryan S, Wechtler H	Journal of Humanitarian Logistics and Supply Chain Management	2021	Proposing a research agenda based on a review of current knowledge and knowledge gaps on the role of collaboration in healthcare supply chains in maintaining optimal stock levels and reinforcing resilience against stockout disruptions during pandemics	Australia, United States of America	Systematic literature review	Relational view theory, risk homeostasis theory	Healthcare		x					x	
Fu HP, Chao P, Chang TH, Chiou CH	Internet Research	2006	Proposing an integrated collaborative website among government agencies to enhance customer satisfaction with the service quality of government agencies	Taiwan – Republic of China	Case study	Collaboration theory	Public services generally		x	x					x
Getele GK, Li T, Arrive JT	IEEE Engineering Management Review	2020	To investigate supply chain management in healthcare service quality in developing countries	China	Questionnaire	Organisational theory	Healthcare	x					x		
Gianakis G, McCue C	Journal of Public Procurement	2012	To examine the factors that either foster or impede the adoption of supply chain management processes by public sector organisations, as well as to identify the potential benefits that local governments can achieve through the use of the supply chain framework	United States of America	Case study	Organisational theory	Public services generally				x		x		x
Harland C, Telgen J, Callender G, Grimm R, Patrucco A	Journal of Supply Chain Management	2019	Examines the role of public procurement as a lever of government policy implementation	Italy, The Netherlands, Australia, United States of America	Case studies	Policy feedback theory	Public services generally				x	x			
Heaslip G, Kovács G	The International Journal of Logistics Management	2019	To explore service triads in humanitarian logistics; to understand the dynamics between principal(s) and agent(s) and how contractual arrangements influence the service buyer–service provider alignment in humanitarian service triads	Ireland, Finland	Case study	Agency theory	Humanitarian aid		x		x				
Hines P, Martins AL, Beale J	Public Money and Management	2008	Explore how Lean Thinking can be successfully extended into the legal sector, which in most advanced economies is dominated by the public sector	United Kingdom, Portugal	Case studies	Leanness theory	Public services generally	x						x	
Hossain MK, Thakur V, Mangla SK	Journal of Business and Industrial Marketing	2021	To propose a hierarchical structural model of enablers of HCSC in the COVID-19 outbreak and identify inter-relationships among them in the healthcare market	India, United Kingdom	Literature review, experts’ opinions	Emergency management	Healthcare		x	x	x				
Kaupa F, Naude MJ	Journal of Global Operations and Strategic Sourcing	2021	To investigate the critical success factors (CSFs) in the supply chain management of essential medicines in the public healthcare delivery system in Malawi	South Africa	Semi-structured interviews, questionnaire	Organisational theory	Healthcare		x		x		x		x
Kebriyaii O, Hamzehei M, Khalilzadeh M	Journal of Humanitarian Logistics and Supply Chain Management	2021	Considering disaster network design under uncertainty and the management of emergency relief volunteers simultaneously	Iran, Peru	Case study	Fuzzy set theory	Disaster relief	x	x						
Kovács G, Matopoulos A, Hayes O	International Journal of Logistics Research and Applications	2010	To examine the benefits and challenges of a beneficiary focus, and in particular, of community-based approaches, for supply chain design in reconstruction	Finland, Greece, Ireland	Case study	Disaster relief	Humanitarian aid	x	x			x			
Kritchanchai D	Operations And Supply Chain Management-An International Journal	2012	To review problems that occurred in healthcare area regarding supply chain management and identify challenges	Thailand	Case study	Organisational theory	Healthcare	x	x						x
Kunz N, Van Wassenhove LN, Besiou M, Hambye C, Kovács G	International Journal of Operations & Production Management	2017	To identify barriers to relevant research in humanitarian logistics	United States of America, France, Germany, Switzerland, Finland	Panel discussion	Humanitarian relief theory	Humanitarian aid		x				x		x
L’Hermitte C, Bowles M, Tatham P, Brooks B	Journal of Humanitarian Logistics and Supply Chain Management	2015	To propose a comprehensive model of the concept of agility in a humanitarian logistics context and to generate a research agenda to test and operationalise this model	Australia	Literature review	Dynamic capabilities theory	Humanitarian aid		x					x	
Lillrank P, Groop J, Venesmaa J	Supply Chain Management	2011	to explore different units of analysis applicable to the analysis of healthcare service supply chains	Finland	Case study	Value co-creation theory	Healthcare	x			x				
Mafini C	Problems and Perspectives in Management	2016	investigation of barriers to the implementation of public supply chain management strategy in the South African public sector	South Africa	Questionnaire	Organisational theory	Public services generally		x				x		
Marques L, Martins M, Araújo C	Production Planning and Control	2020	mapping recent studies addressing supply chain management (SCM) in the healthcare sector	Brasil	Literature review	Network theory	Healthcare	x	x						
McAdam R, Hazlett S, Johnston S	International Journal of Public Sector Management	2011	to explore the formative development of construction supply chain guidelines or proposals in a UK region’s schools’ estates procurement process to more effectively address a forthcoming increase in investment	United Kingdom	Semi‐structured interviews, focus groups	Organisational theory	Public services generally		x		x				
McKone-Sweet KE, Hamilton P, Willis SB	Journal of Supply Chain Managemen	2005	to explore the barriers to the implementation of SCM practices within the healthcare industry	United States of America	Literature review, case studies	Organisational culture and change theories	Healthcare		x		x		x		
McLachlin R, Larson PD	Journal of Humanitarian Logistics and Supply Chain Management	2011	to advance thought and practice on supply chain relationship building in the context of humanitarian logistics, drawing on lessons from leading practitioners	Canada	Experts opinion	Relationship theory	Humanitarian aid		x		x				
Meijboom B, Schmidt‐Bakx S, Westert G	Supply Chain Management	2011	to discuss organisational problems that occur in situations that are complex because the treatment of patients requires input from multiple health care providers, and to argue how to resolve these problems by using SCM practices	The Netherlands	Literature review	Service theory	Healthcare	x	x						
Migiro SO, Ambe IM	African Journal of Business Management	2008	evaluating whether and to what extent supply chain management (SCM) practices in local municipalities are implemented	South Africa	Case study	Contingency and force field theories	Public services generally	x	x		x				
Nagurney A, Flores EA, Soylu C	Transportation Research Part E: Logistics and Transportation Review	2016	to develop a Generalised Nash Equilibrium network model for post-disaster humanitarian relief by nongovernmental organisations	United States of America	Case study	Game theory	Humanitarian aid	x		x	x				
Namagembe S	Journal of Humanitarian Logistics and Supply Chain Management	2020	examination of the influence of relational capital on inter-cluster coordination and service delivery of humanitarian organisations	Uganda	Survey	Resource-based view theory	Humanitarian aid			x	x	x			
Nayak R, Choudhary S	Production Planning and Control	2020	present results of a research on how a novel integrated lean and agile (leagile) framework could be employed to efficiently and effectively manage humanitarian logistics and supply chain management (HLSCM) in a local jurisdiction (i.e. public sector) of a disaster-hit region in a non-mature economy	United Kingdom	In-depth case study	Operational efficiency theory	Humanitarian aid	x		x	x			x	
Nelan MM, Wachtendorf T, Penta S	Risk, Hazards and Crisis in Public Policy	2018	to examine how involved in donation drives come to make sense of agility’s necessity in disaster supply chains	United States of America	Interview	Social constructivist paradigm	Disaster relief			x		x		x	x
Obaze Y	Journal of Humanitarian Logistics and Supply Chain Management	2019	to explore the humanitarian service management categories that influence long-term transformation within complex community-based service ecosystems	United States of America	Case study	Service-dominant logic and service science theories	Humanitarian aid	x	x		x	x			
Okdinawati L, Dwiputranti MI, Oktora RA	International Journal of Emergency Management	2019	to develop the coordination structure as a joint effort in humanitarian relief operations	Indonesia	Case study	Coordination theory	Humanitarian aid		x	x					x
Oloruntoba R, Gray R	Supply Chain Management	2006	to investigate the nature of the humanitarian aid supply chain and discuss the extent to which certain business supply chain concepts, particularly supply chain agility, are relevant to humanitarian aid	Australia, United Kingdom	Case studies	Agility theory	Humanitarian aid	x	x					x	
Oloruntoba R, Kovács G	Supply Chain Management	2015	to provide a commentary and an overview of developments in the field of humanitarianism that could impact theoretical understanding of agility in humanitarian aid supply chains over the past decade	Australia, Finland	Literature review	Agility theory	Humanitarian aid	x		x				x	
Ozdemir AI, Erol I, Ar IM, Peker I, Asgary A, Medeni TD, Medeni IT	The International Journal of Logistics Management	2021	to investigate the role of blockchain in reducing the impact of barriers to humanitarian supply chain management (HSCM) using a list of blockchain benefits	Turkey, Canada	interview with experts	fuzzy set theory	humanitarian aid		x						x
Pettit S, Beresford A	International Journal of Physical Distribution & Logistics Management	2009	to set out the key areas for research in the context of humanitarian aid	United Kingdom	Literature review	Organisational theory	Humanitarian aid		x					x	x
Pettit S, Beresford A, Knight D, Sohn M	Public service operations management: a research handbook	2016	to highlight the logistics challenges involved in responses to natural disasters and to analyse the applicability of commercial logistics/supply chain management (SCM) concepts in humanitarian situations	United Kingdom, Finland	Literature review	Agility and leanness theories	Humanitarian aid	x						x	x
Pillitteri F, Mazzola E, Bruccoleri M	Journal of Humanitarian Logistics and Supply Chain Management	2021	analysing value co-creation processes in humanitarian professional services provision and the key enabling factors of beneficiaries’ participation involved in long-term integration programmes	Italy	Case study	Value co-creation theory	Humanitarian aid		x			x	x		
Polater A, Demirdogen O	International Journal of Pharmaceutical and Healthcare Marketing	2018	to focus on the impact of supply chain (SC) integration, demand forecasting and supplier performance on patient responsiveness at public hospitals through the mediating role of SC flexibility	Turkey	Questionnaire	Network and the resource-based view theories	Healthcare	x	x				x		
Quarshie AM, Leuschner R	Journal of Supply Chain Management	2020	to study inter-organisational interaction in the context of a major US disaster: Hurricane Sandy	Finland, United States of America	Case study	Inductive grounded theory	Disaster relief	x	x	x	x				
Radnor ZJ, Noke H	Production Planning and Control	2013	to conceptualise and contextualise operations management within the public sector	United Kingdom	Literature review	Operations management theory	Public services generally		x				x		
Sampson SE, Schmidt G, Gardner JW, Van Orden J	Journal of Business Logistics	2015	to build an ex-post theory of coordination in health care service supply networks using process-based analysis of entity interactions	United States of America	Case study	Coordination theory	Healthcare	x		x					
Scala B, Lindsay CF	Supply Chain Management	2021	to explore how resilience is evident in healthcare supply chains in the public sector when faced with pandemic disruption and to identify any learnings to inform recovery and future-readiness phases	United Kingdom	Caser study	Resilience theory	Healthcare		x					x	
Scholten K, Scott PS, Fynes B	International Journal of Physical Distribution and Logistics Management	2010	to explore the concept of agility in the context of supply chains of humanitarian aid (HA) organisations, particularly non-government organisations (NGOs)	Ireland	Literature review, semi-structured interviews	Agility theory	Humanitarian aid							x	x
Seepma AP, de Blok C, Van Donk DP	Supply Chain Management	2020	to explore how inter-organisational ICT is used in redesigning public service supply chains	The Netherlands	Multiple-case study	Inter-organisational perspective	Public services generally	x	x	x					
Seepma AP, Van Donk DP, de Blok C	Journal of Supply Chain Management	2021	theory elaboration on the applicability of for-profit management approaches in public contexts and supply chains	The Netherlands	Case study	Publicness theory	Public services generally	x							x
Shafiq M, Soratana K	Journal of Humanitarian Logistics and Supply Chain Management	2020	to review previous studies and identify areas needing further improvements in Humanitarian Organizations Logistics and Supply Chain Management (HO-LSCM) system	Pakistan, Thailand	Literature review	Leanness theory	Humanitarian aid		x					x	
Shafiq M, Soratana K	Logforum	2019	to present a Lean Readiness Assessment Model (LRAM) for assessing the readiness of Humanitarian Organisations (HO) to adopt Lean Management (LM) practices	Thailand	Questionnaire survey	Disaster relief	Humanitarian aid		x	x				x	
Stewart GT, Kolluru R, Smith M	International Journal of Physical Distribution & Logistics Management	2009	to sharpen the concept of national resilience by recommending a framework that positions community resilience as an integral variable in understanding the ability of impacted areas to manage the consequences of disasters effectively	United States of America	Literature review	Resource advantage theory	Disaster relief		x		x			x	
Szoltysek J, Fraczkiewicz-Wronka A	Management	2012	evaluation of the effectiveness of social service organisations	Poland	Literature review	Network theory	Public services generally			x	x				
